# Building a Barrier: The Influence of Different Wax Fractions on the Water Transpiration Barrier of Leaf Cuticles

**DOI:** 10.3389/fpls.2021.766602

**Published:** 2022-01-05

**Authors:** Pascal Seufert, Simona Staiger, Katja Arand, Amauri Bueno, Markus Burghardt, Markus Riederer

**Affiliations:** Chair of Botany II – Ecophysiology and Vegetation Ecology, Julius von Sachs Institute of Biological Sciences, University of Würzburg, Würzburg, Germany

**Keywords:** leaf cuticles, non-stomatal transpiration, selective wax extraction, triterpenoids, very long-chain aliphatics, weighted average chain length, leaf cuticular wax properties

## Abstract

Waxes are critical in limiting non-stomatal water loss in higher terrestrial plants by making up the limiting barrier for water diffusion across cuticles. Using a differential extraction protocol, we investigated the influence of various wax fractions on the cuticular transpiration barrier. Triterpenoids (TRPs) and very long-chain aliphatics (VLCAs) were selectively extracted from isolated adaxial leaf cuticles using methanol (MeOH) followed by chloroform (TCM). The water permeabilities of the native and the solvent-treated cuticles were measured gravimetrically. Seven plant species (*Camellia sinensis*, *Ficus elastica*, *Hedera helix*, *Ilex aquifolium*, *Nerium oleander*, *Vinca minor,* and *Zamioculcas zamiifolia*) with highly varying wax compositions ranging from nearly pure VLCA- to TRP-dominated waxes were selected. After TRP removal with MeOH, water permeability did not or only slightly increase. The subsequent VLCA extraction with TCM led to increases in cuticular water permeabilities by up to two orders of magnitude. These effects were consistent across all species investigated, providing direct evidence that the cuticular transpiration barrier is mainly composed of VLCA. In contrast, TRPs play no or only a minor role in controlling water loss.

## Introduction

Plant waxes are complex mixtures varying across species. They are heterogeneous mixtures with more than a hundred compounds, sometimes dominated by a single compound by up to 70%. These complex blends have evolved to fulfill various biological functions, like protection against UV radiation, fungi and insects, self-cleaning of the surface, and building an uptake barrier (Yeats and Rose, 2013). The most important function is to limit cuticular transpiration, especially during periods of drought. It is well known that cuticular waxes build the main barrier to non-stomatal water loss across the leaf surface since the extraction of the cuticular wax leads to a drastic increase of cuticular water permeability (Schönherr, 1976; Schönherr and Lendzian, 1981). However, what wax traits are responsible for its barrier properties remain unclear.

Contrary to intuitive expectation and the statements in many plant ecology text books, the water permeability of cuticles is neither correlated to their thickness (Kamp, 1930; Evans, 1932; Schönherr, 1976, 1982; Becker et al., 1986; Schreiber and Riederer, 1996; Riederer and Schreiber, 2001; Burghardt and Riederer, 2006) nor to total wax, intracuticular wax, or epicuticular wax coverage (Riederer and Schneider, 1990; Riederer and Schreiber, 2001; Jetter and Riederer, 2016; Bueno et al., 2020). Hence, after more than one century of research into the ecophysiology of cuticular transpiration, the molecular mechanism underlying the barrier properties of plant cuticles is still largely unknown. This could be because the scientists in this field asked the wrong questions and, therefore, got just such answers. The present work aims to clarify what we have to understand by “wax” when we want to elucidate the chemical and physical foundations of the cuticular transpiration barrier.

The composition of leaf cuticular waxes differs widely between plant species, but alicyclic triterpenoids (TRPs) and very long-chain aliphatics (VLCAs) are usually the most abundant constituents (Jetter et al., 2006; Busta and Reinhard, 2018). In the adaxial leaf wax of *Prunus laurocerasus*, the former constitutes about 70% of the total wax coverage. Although VLCAs make up a significant wax fraction in many leaf cuticles analyzed so far, TRPs can nevertheless constitute the major wax fraction. Within the VLCAs, alkanes, primary alcohols, and fatty acids are usually the most abundant substance classes. In several species, a significant difference regarding their chemical composition between intra- and epicuticular waxes was found. While epicuticular wax only contains aliphatic compounds, intracuticular wax comprises both TRPs and VLCAs (Jetter et al., 2000; Jetter and Riederer, 2016; Zeisler and Schreiber, 2016). Several studies have shown that the transpiration barrier is located within the intracuticular wax in most plant species, embedded in the cutin matrix (Jetter and Riederer, 2016; Zeisler and Schreiber, 2016; Zeisler-Diehl et al., 2018). Jetter and Riederer (2016) proposed that the transpiration barrier is mainly formed by the intracuticular VLCAs, while alicyclic TRPs play no or only a minor role. Other studies comparing the wax composition of genetically modified plants (Vogg et al., 2004) or intra- and epicuticular wax of the same cuticle (Jetter and Riederer, 2016) also suggested that the VLCAs mainly constitute the cuticular transpiration barrier. VLCAs at physiological temperatures form semi-crystalline solids (Merk et al., 1998; Dorset, 2005; Fagerström et al., 2013) with crystalline domains consisting of the tightly aligned long hydrocarbon chains of the aliphatic compounds (Reynhardt and Riederer, 1991, 1994; Dorset, 1999) and having a low permeability for water. TRPs have been shown to provide mechanical (Tsubaki et al., 2013) and thermal stability to the plant cuticle (Schuster et al., 2016).

The present study aims to shed light on the roles of the VLCA and TRP fractions in plant cuticular waxes in building the barrier against the transcuticular diffusion of water. A suite of seven plant species was selected for the experiments, which fulfilled the following conditions: (1) astomatous leaf cuticles can be isolated, (2) presence of TRPs varying from absent to making up high proportions, and (3) absence of pronounced epicuticular wax crystals. To examine the respective impact of the two wax fractions on the permeability properties of the cuticle, TRPs and VLCAs were selectively removed by solvent extraction of isolated cuticles. Permeances for water were obtained for (1) native isolated cuticular membranes (CMs) containing both the VLCA fraction and if present, the TRP fraction of cuticular wax, (2) MeOH-extracted cuticular membranes (Ms) containing the VLCA fraction but TRPs having been selectively removed, and (3) chloroform-extracted and thus completely dewaxed matrix membranes (MXs). This approach was taken to clarify which wax fractions we must keep in mind when we want to understand cuticular barrier properties.

## Materials and Methods

### Plant Material and Cuticle Isolation

Plants of *Vinca minor*, *Hedera helix*, and *Ilex aquifolium* were grown outdoors in the Botanical Garden of the University of Würzburg. *Ficus elastica*, *Camellia sinensis*, and *Zamioculcas zamiifolia* were cultivated in the greenhouse all year round. At the same time, potted *Nerium oleander* plants were held in the greenhouse during winter and outdoors during summer.

Astomatous CMs were isolated according to a method adapted from Schönherr and Riederer (1986). For all plants except *V. minor*, disks with a diameter of 1.89 cm were punched out of fresh, fully mature leaves, avoiding major veins as far as possible. From the tiny leaves of *V. minor,* disks with a diameter of only 1.66 cm were punched out, and it was impossible to avoid the major vein. After marking the adaxial, astomatous side at the edge of the leaf disks with a permanent marker pen, the disks were incubated in an enzyme solution containing 1% pectinase (Trenolin, Erbslöh, Geisenheim, Germany), 1% cellulase (Celluclast, NCBE, University of Reading, U.K.), 1 mm citric acid monohydrate (Applichem, Darmstadt, Germany) as a buffer, and 1 mm sodium azide (Sigma-Aldrich, Steinheim, Germany) to prevent bacterial growth. The solution was kept at room temperature and was replaced twice a week. After the disintegration of the leaf tissue, astomatous cuticles (CM) were identified by the pen marks, washed thoroughly with distilled water and stored in distilled water until further use (maximum period of 7 weeks).

### Wax Extraction and Analysis

For full extracts (FE), samples comprised of five CMs per plant species (two for *H. helix*) were extracted twice, first overnight and then for 30 min, in 10 ml TCM (≥99.8%; Roth, Karlsruhe, Germany) at room temperature, yielding completely dewaxed matrix membranes (MX). N-tetracosane (Sigma-Aldrich, Steinheim, Germany) was added as an internal standard before the first extraction. TCM extracts were combined, and the solvent was evaporated under a gentle stream of N_2_. Derivatization for gas chromatography of an aliquot of the wax samples was performed using N,O-bis (trimethylsilyl) trifluoroacetamide (Macherey-Nagel Düren, Germany) in dry pyridine (Roth, Karlsruhe, Germany) for 45 min at 70°C. Quantitative analyses were performed using a gas chromatograph coupled with a flame ionization detector (FID). Samples were injected by an on-column injector (7890A, Agilent Technologies, Waldbronn, Germany) into a fused silica capillary column (DB1-ms, 30 m length×0.32 mm ID, 0.1 μm film, Agilent Technologies) using H_2_ as the carrier gas. The temperature program was as: injection at 50°C and held for 2 min, raised by 40°C min^−1^ to 200°C and held for 2 min, and increased by 3°C min^−1^ to 320°C and held for 30 min. Quantification was achieved by comparing the internal standard’s peak area with the analytes’ peak area. Gas chromatography coupled with mass spectrometry (5975iMSD, Agilent Technologies) was used for qualitative analysis. The separation was performed under the same conditions except that helium was used as the carrier gas.

For selective wax extraction, five CMs per sample were extracted overnight with 20 ml MeOH (UPLC-grade; Roth, Karlsruhe, Germany) at room temperature. Depending on the plant species, four to eight samples were prepared. The cuticles were removed and washed with MeOH. The extracts and washings were combined. Part of the MeOH-extracted cuticles (Ms) were saved for permeability measurements. The remaining Ms were gently flattened, dried under a stream of air on Teflon platelets, and immersed twice, first overnight, then for 30 min in 20 ml TCM (≥99.8%; Roth, Karlsruhe, Germany) at room temperature to ensure complete wax removal. This extraction process yielded dewaxed matrix membranes (MXs) equivalent to the MXs obtained by TCM extraction of CMs. The MXs were washed with TCM, flattened on Teflon platelets, and dried under a gentle stream of air. TCM extracts and washings were combined, and n-tetracosane was added as an internal standard. Wax analysis was performed as described above.

### Measurement of Cuticular Permeance

Permeances of CMs, Ms, and MXs were measured by a method adapted from Schönherr and Lendzian (1981). The cuticles were mounted on stainless steel transpiration chambers using Teflon paste (Roth, Karlsruhe, Germany) and fixed with a ring-like lid. The physiological outer side of the isolated cuticles faced the atmosphere with a surface area of 1.12 cm^2^ available for transpiration. After adding deionized water through a small opening in the bottom of the transpiration chamber, the latter was sealed with adhesive tape. The transpiration chambers were placed into a sealed plastic container upside down on a grid over dried silica gel (Applichem, Darmstadt, Germany). The chambers were kept at 25°C. The transpirational water loss was determined gravimetrically. Samples were weighted once or twice a day, depending on the plant species and treatment with a total of 6 measurements. Water permeance P (m s^−1^) was obtained from


(1) $$P=\frac{J}{A\times \Delta c}$$

with J being the loss of water (g s^−1^), A the cuticle surface area exposed to the air (m^2^), and Δc the difference of water vapor concentration at the inner (C_i_ = 23.074 g m^−3^) and outer (C_o_ = 0 g m^−3^) sides of the cuticle.

### Scanning Electron Microscopy

The surface structure of the outer sides of the isolated cuticles was investigated using scanning electron microscopy (SEM, JEOL JSM-7500F, JEOL GmbH, Freising, Germany) with an accelerating voltage of 5.0 kV and a working distance of 7.9 mm equipped with a field emission gun and a lower secondary electron (LEI) detector. CMs, Ms, and MXs were carefully mounted on aluminum holders and sputter-coated with an 80/20 gold/palladium mixture.

### Statistics

OriginPro 2018b (Systat Software GmbH) was used for statistical analysis. Water permeance values and their log transformations were rejected for normal distribution with the Shapiro–Wilk normality test. Therefore, medians (median; 25th–75th percentile) and nonparametric statistics were used. Permeances were tested for significant differences by Kruskal-Wallis test ANOVA with post-hoc Dunn’s test (*p* < 0.05). For wax analyses, normal distribution was found for all species except for the VLCAs of the MeOH and TCM extracts of *V. minor* and the VLCAs of the MeOH extract of *Z. zamiifolia*. For normally distributed data, significant differences were investigated using one-way ANOVA (*p* < 0.05). Otherwise, the Kruskal-Wallis test ANOVA with post-hoc Dunn’s test was used.

## Results

### Cuticular Wax

**Figure 1:**
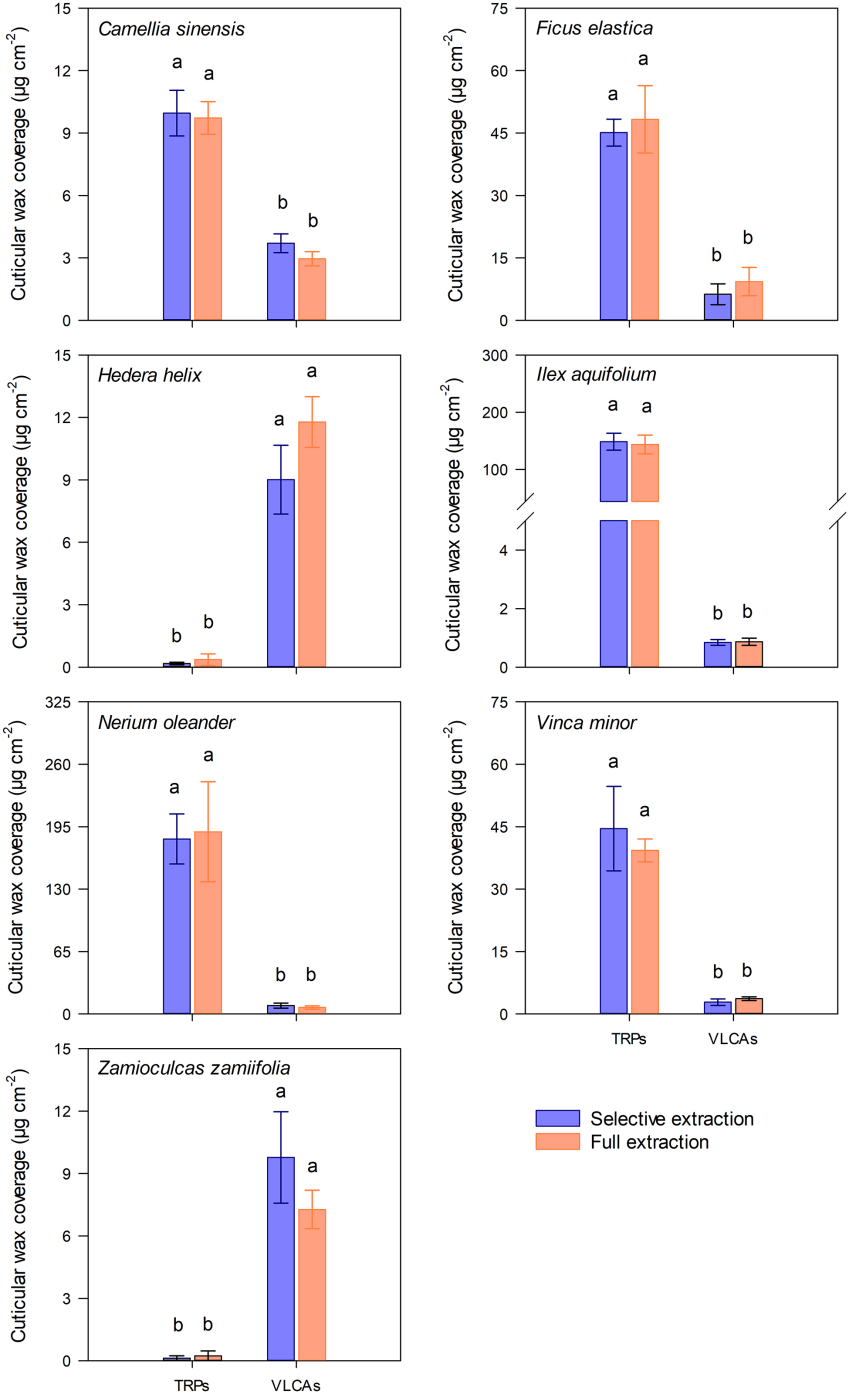
Comparison of total cuticular loads of very long-chain aliphatics (VLCA) and triterpenoids (TRP) in selective wax extracts (purple) and full extracts (orange) of seven plant species. Data were normally distributed. Differences were tested using one-way ANOVA. Different letters stand for significant differences (*p* < 0.05) between the two extraction methods within a wax fraction. Columns show mean values and whiskers standard deviations (*n* = 4–8).

A complex mixture of cuticular waxes was found in MeOH and following TCM extracts, and full TCM extracts of cuticles from the seven plant species investigated (Supplementary Table 1). *Z. zamiifolia* and *H. helix* had low total wax coverages (around 10 μg cm^−2^), consisting almost exclusively of VLCAs. *C. sinensis* showed a wax composition of about one-third of VLCAs and two-thirds of TRPs. *V. minor* and *F. elastica* had intermediate wax coverages (40–60 μg cm^−2^) with minor VLCA content. *N. oleander* and *I. aquifolium* exhibited high wax coverages (around 180 μg cm^−2^) dominated by TRPs and only minor contributions of VLCAs (Supplementary Table 2). No significant differences of the total removed TRP and VLCA wax coverages between the combined MeOH and following TCM extracts and FEs were observed (Figure 1).

**Figure 2:**
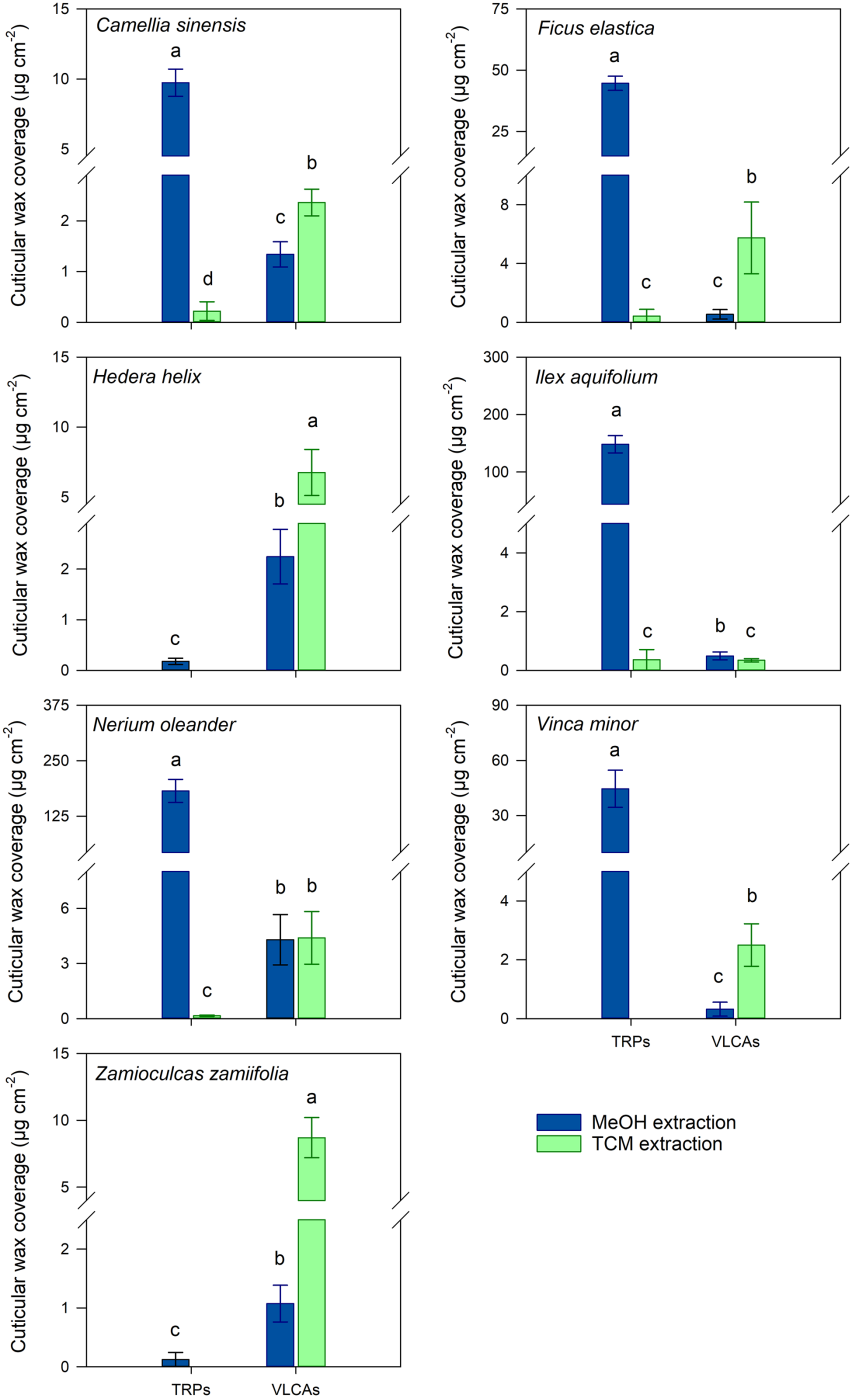
Comparison of cuticular wax compositions extracted from isolated cuticles with consecutive methanol (MeOH; blue) and chloroform (TCM; green) extraction steps. Normal distribution was found for data of all species except for the VLCA of both steps of *V. minor* and the VLCAs of the MeOH extraction of *Z. zamiifolia*. For normally distributed data, differences were tested for significance with one-way ANOVA. In all other cases, the Kruskal-Wallis test ANOVA with post-hoc Dunn’s test was used. Different letters stand for significant differences (*p* < 0.05) between the two extraction methods within a wax fraction. Columns show mean values and whiskers standard deviation (*n* = 4–8).

MeOH treatment removed TRPs exhaustively or almost exhaustively in all TRP-containing species. In most species, VLCA in the MeOH extract ranged from 11 to 44% of the total extracted VLCAs. Only from *I. aquifolium*, about 60% of the VLCAs were removed with MeOH (Supplementary Table 2). Subsequent TCM treatment of these cuticles released almost exclusively VLCAs and minor residues of TRPs (Figure 2; Supplementary Table 2). All plant species showed a significant decrease of extracted TRPs between the MeOH and the following TCM extract (*p* < 0.05). Except for *I. aquifolium* and *N. oleander*, all plant species showed a significant increase of extracted VLCAs between the MeOH and the following TCM extract (*p* < 0.05). Despite considerable differences in wax coverages and compositions, these trends were observed for all plant species (Supplementary Table 2).

**Figure 3:**
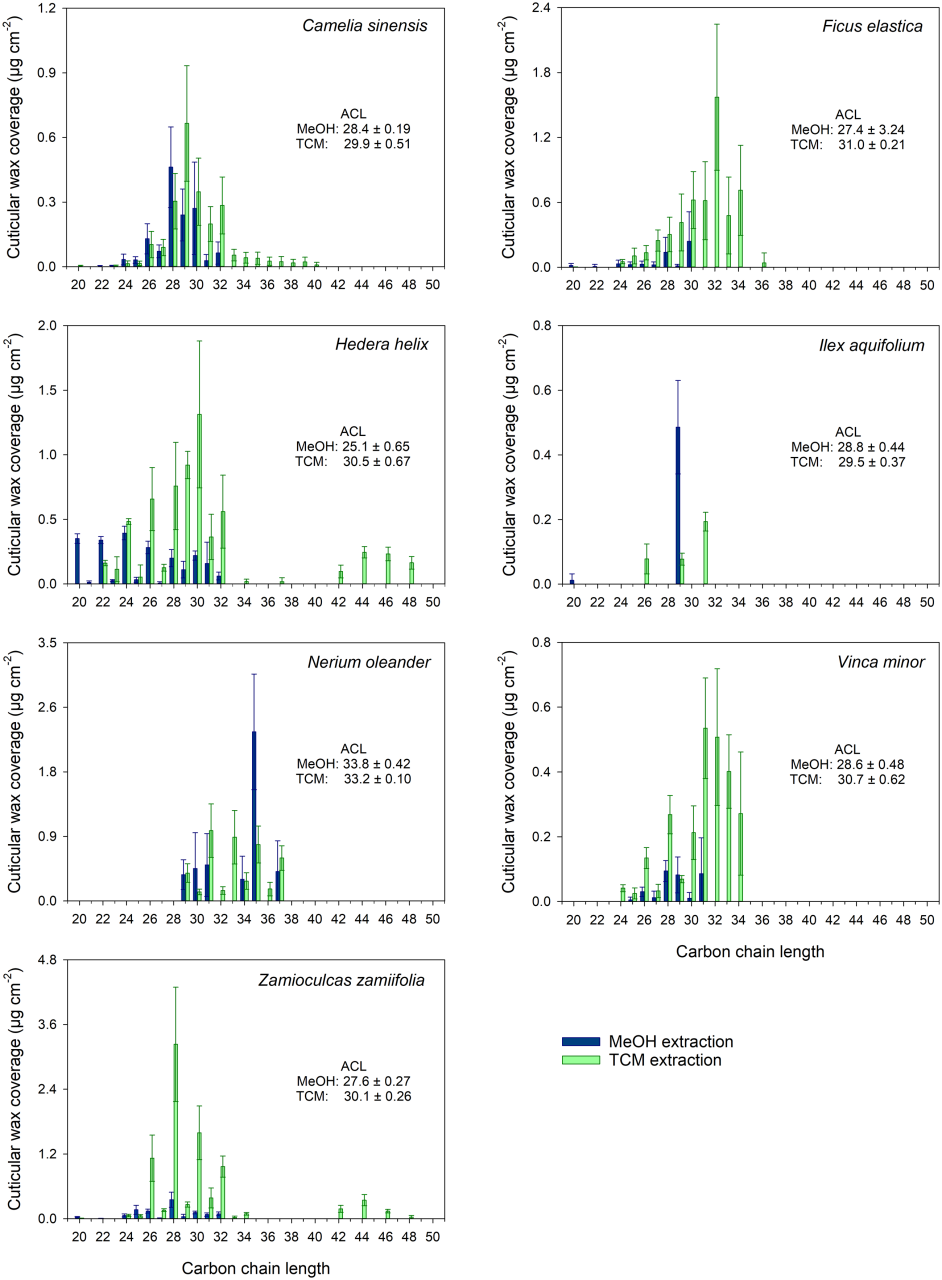
Chain length distribution of VLCA in the methanol (MeOH; blue) and the subsequent chloroform (TCM; green) extracts of seven plant species. Columns show mean values and whiskers standard deviation (*n* = 4–8).

The chain length distribution of the VLCAs varied strongly between the plant species. In all plant species, except *N. oleander,* the MeOH extraction tended to remove VLCAs with shorter chain lengths (< 32 carbon atoms). In comparison, the following TCM extraction also removed VLCAs with longer chain lengths (Figure 3).

### Water Permeance

**Figure 4:**
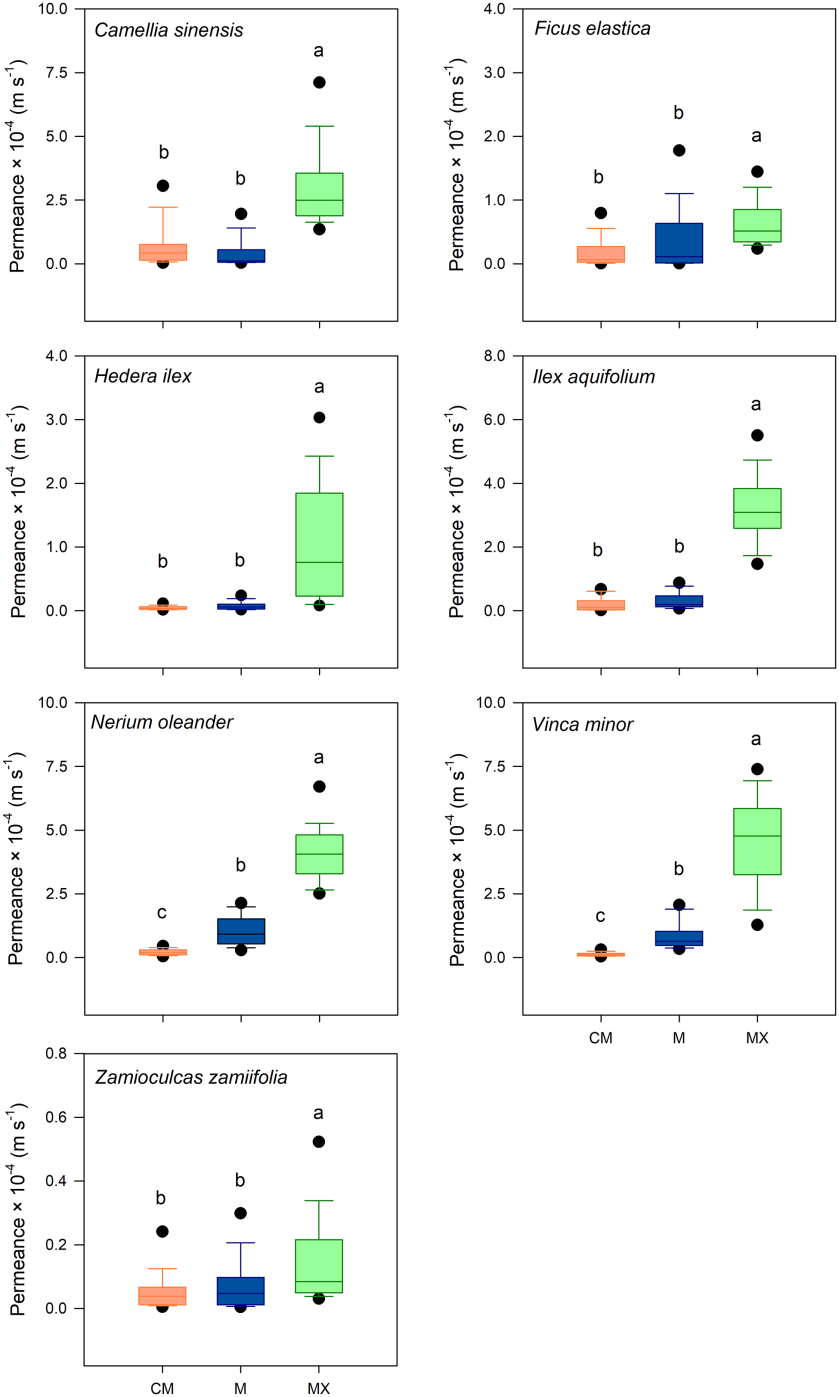
Water permeability of isolated cuticles of seven plant species in untreated (CM; orange), methanol (M; blue), and subsequent chloroform (MX; green) extracted conditions. Investigation of significant differences was conducted by Kruskal-Wallis test ANOVA with post-hoc Dunn’s test. Boxes are interquartile ranges from 25 to 75%, whiskers 10–90% and dots designate outliers. Different letters stand for significant differences (*p* < 0.05) among treatments (*p* ≥ 28).

The cuticular water permeance was measured for CMs, Ms, and MXs (Supplementary Table 3). Median permeances of CMs ranged from 0.40 × 10^−5^ m s^−1^ (*H. helix*) to 4.27 × 10^−5^ m s^−1^ (*C. sinensis*). Permeances of Ms ranged from 0.57 × 10^−5^ m s^−1^ (*Z. zamiifolia*) to 9.19 × 10^−5^ m s^−1^ (*N. oleander*). The permeances of Ms of *C. sinensis*, *F. elastica*, *H. helix*, *I. aquifolium,* and *Z. zamiifolia* were not significantly different (*p* > 0.05) from the values of the untreated cuticles (Figure 4). Permeances of Ms of *N. oleander* and *V. minor* showed a small but significant increase (*p* < 0.05) in comparison with the values of CMs (Figure 4). Permeances of MXs ranged from 1.07 × 10^−5^ m s^−1^ (*Z. zamiifolia*) to 50.9 × 10^−5^ m s^−1^ (*V. minor*). The permeances of the completely dewaxed MXs of the species investigated were significantly higher (*p* < 0.05) than those of CMs and Ms (Figure 4).

### Scanning Electron Microscopy

The surfaces of CMs of all species investigated were smooth, lacking pronounced and well-defined epicuticular wax crystals (Supplementary Figures 1–4). The surface of *C. sinensis* displayed tiny, scattered wax accumulations. The *F. elastica* surface appeared encrusted. Ms of both plant species showed a slight decrease in surface roughness. (Supplementary Figure 1). Aside from small wax accumulations, the surfaces of *H. helix* and *I. aquifolium* had an overall smooth appearance. The M of *I. aquifolium* showed relatively small differences to the corresponding CM. The M of *H. helix* appeared slightly more granulated than the corresponding CM. (Supplementary Figure 2). The wax on the surface of CM and M of *N. oleander* had a smooth appearance. The CM and M of *V. minor* were encrusted with wax (Supplementary Figure 3). The surface CM and M of *Z. zamiifolia* had a partially granulated appearance. (Supplementary Figure 4). Aside from minor remnants, the surfaces of MXs of all plants appeared devoid of cuticular wax (Supplementary Figures 1–4).

## Discussion

The objective of our study was to experimentally identify the respective contributions of the two main cuticular wax fractions VLCAs and TRPs to the barrier against non-stomatal water loss. The effects of the complete and the selective removal of TRPs and VLCAs, respectively, on the water permeability barrier were measured using isolated cuticles from seven plant species with a varying contribution of TRPs to the total of their cuticular waxes.

The primary challenge was to find a solvent suitable for TRP extraction, which leaves the VLCA fraction of the cuticular wax as intact as possible. Reports dealing with the solubility of VLCA compounds in organic solvents indicate a common trend of a sharply decreasing solubility of these substances with increasing chain lengths (Hoerr et al., 1944; Kiser et al., 1961). Hoerr et al. (1944) showed this trend in several solvents varying in polarity for primary *n*-alcohols with even carbon chain lengths between 10 and 18. A similar result was found for the solubility of hydrocarbons with chain lengths between five and 10 in MeOH (Kiser et al., 1961). Riederer and Schneider (1989) recalculated data from several publications and reported the relative solubility compared to their solubility in TCM of three substances found in cuticular wax. TCM was by far the best solvent for both 1-octacosanol and octadecanoic acid. However, both compounds were only slightly soluble in MeOH, and the solubility of dotriacontane was higher in more nonpolar solvents (Riederer and Schneider, 1989). Jin et al. (1997) investigated the solubility of oleanolic and ursolic acid in different solvents, and their solubility in MeOH was similar to that in TCM. From the present data, assuming equal accessibility of the wax components for the solvent, we expected a good solubility of TRPs in MeOH but not of VLCAs, allowing us to extract the two fractions separately from the cuticle.

Treating isolated cuticles containing a TRP wax fraction with MeOH led to almost complete removal of this distinctly polar alicyclic fraction. In addition to TRPs, minor amounts of the more polar VLCAs were released, mainly those with shorter chain lengths and polar functional groups, like alcohols and fatty acids. Most importantly, the majority of the VLCAs with higher carbon numbers remained in the cuticle. They could be extracted with a subsequent TCM treatment (Figure 2). Despite a relatively poor solubility of VLCAs in MeOH, a significant fraction of the total VLCA amount was extracted in *I. aquifolium* and *N. oleander*. These results show that our method could remove TRPs quite selectively, however, not without avoiding VLCA removal. This may indicate that in some species, TRPs and VLCAs are not as distinctly separated in different layers as postulated by Jetter and Riederer (2016) but may intermix to some degree. It is conceivable that a portion of VLCAs is extracted by MeOH when the surrounding TRPs are removed, and the connection to the cutin matrix gets lost. The chain length distributions of VLCAs extracted with MeOH and TCM differed strongly. MeOH dissolved VLCAs preferentially with shorter chain lengths (Figure 3), while TCM did not discriminate chain lengths. VLCAs with a polar functional group dominated the long-chain fraction of the MeOH extracts, which also contained alkanes to a minor extent. VLCAs with longer chain lengths, including alkyl esters with carbon numbers above 40, could be found in the following TCM extract. VLCAs were extracted exhaustively using TCM, regardless their functional groups or chain lengths.

To characterize the VLCAs extracted from CMs with MeOH and from Ms with TCM, we calculated the weighted average chain length (ACL) as an indicator for the ability of the solvent to dissolve VLCAs with varying carbon numbers. ACLs were calculated according to:


(2) $$ACL=\frac{\sum \left(C_{n}\times n\right)}{\sum \left(C_{n}\right)}$$

where c_n_ is the amount per unit area of all aliphatic compounds with carbon chain length n. The chain length distribution of the MeOH extracts was characterized by an ACL < 30. *N. oleander* was the only species with an ACL > 30 in the MeOH extract. The TCM extracts from Ms showed ACLs >30 except those of *C. sinensis* and *I. aquifolium*, which were dominated by shorter chain aliphatics. Combining the results for the two extracts yielded ACLs >30 for *F. elastica, N. oleander*, and *V. minor,* while all other species exhibited ACLs below 30. The same was found for FEs (obtained by TCM extraction of CMs); *F. elastica*, *N. oleander,* and *V. minor* showed ACL values above 30, all other plant species below 30 (Supplementary Table 4). The ACLs of TCM extracts of all plants, except *N. oleander,* displayed a significant increase (*p* < 0.05) compared to the ACLs of MeOH extracts, indicating a reduced VLCA extraction capability of MeOH compared to TCM with the increasing chain length of the wax constituent. The ACLs of the combined MeOH and TCM extracts and FEs did not differ significantly (*p* > 0.05).

Removing the TRPs from CMs with MeOH did not significantly affect the cuticular water permeance of isolated cuticles except for *N. oleander* and *V. minor*. However, the subsequent extraction of the remaining VLCAs with TCM substantially increased the permeances of the cuticles of all plant species compared to untreated and MeOH-extracted ones (Figure 4). These findings are rigorous experimental evidence that the VLCA and not the TRP fraction of cuticular wax constitutes the actual barrier to water permeability. The results for *I. aquifolium*, *N. oleander*, and *V. minor* indicate a more complex relationship between water permeance and the VLCAs, which needs further study. One can be imagined that not only the composition of cuticular waxes influences the barrier properties but also their location and distribution within the cuticle.

Since the MeOH treatment did not alter the integrity of the transpirational barrier made up by the VLCAs in the majority of the species investigated, we propose that the VLCA and TRP wax fractions are located in different regions of the cuticle depending on their polarity with some degree of intermixing. As already shown for several plant species, the VLCAs tend to accumulate at and on the outer surface of the cuticle facing the environment, while TRPs are deposited in the interior of the cuticle, facing the hydrophilic cell wall (Jetter et al., 2000; Buschhaus and Jetter, 2011; Jetter and Riederer, 2016). Jetter and Riederer (2016) postulated that cuticular waxes are arranged in a VLCA layer associated with the outer surface of the cuticle and a TRP layer in its interior. This view was supported by a previous study (Staiger et al., 2019) describing that no TRPs could be extracted when dipping whole leaves into MeOH. This indicates that the external VLCA layer prevents MeOH from penetrating into deeper layers of the cuticle from the outside and extracting TRPs from these locations. Extraction of TRPs with MeOH was only possible with isolated cuticles, whose inner side is fully accessible to the solvent.

Our findings provide, to our knowledge, for the first time direct experimental evidence for the fact that the cuticular transpiration barrier is mainly composed of the VLCA fraction of the wax. This experimental approach makes it possible to more clearly formulate questions concerning the significance of waxes or specific fractions thereof for the cuticular transpiration barrier and to generate hypotheses that can be tested experimentally. This may translate into a significant advance in the scientific understanding of the role of the cuticle in the ecophysiology of non-stomatal transpiration beyond the intuitive but mostly wrong views prevalent in this field.

## Data Availability Statement

The raw data supporting the conclusions of this article will be made available by the authors, without undue reservation.

## Author Contributions

PS and SS conducted the experiments and evaluated the results together with KA. SS, KA, AB, MB, and MR revised the manuscript. PS wrote the manuscript. AB implemented the changes. KA, MB, and MR supervised the work. Results and content were discussed with all authors. All authors contributed to the article and approved the submitted version.

## Conflict of Interest

The authors declare that the research was conducted in the absence of any commercial or financial relationships that could be construed as a potential conflict of interest.

## Publisher’s Note

All claims expressed in this article are solely those of the authors and do not necessarily represent those of their affiliated organizations, or those of the publisher, the editors and the reviewers. Any product that may be evaluated in this article, or claim that may be made by its manufacturer, is not guaranteed or endorsed by the publisher.

## Abbreviations

CM: Cuticular membrane
FE: Full extracts
M: Methanol extracted cuticle
MX: Dewaxed cuticles
MeOH: Methanol
TCM: Chloroform
TRP: Triterpenoid
VLCA: Very long-chain aliphatic
