# Supplementary material for: Building a Barrier: The Influence of Different Wax Fractions on the Water Transpiration Barrier of Leaf Cuticles
Source: Front Plant Sci. 2022 Jan 5;12:766602. doi: 10.3389/fpls.2021.766602 (PMC8766326; doi:10.3389/fpls.2021.766602)
Supplement: Supplementary file 1 [file Data_Sheet_1.PDF]

Supplementary Tables

Supplementary Table 1. Chemical composition of leaf cuticular waxes of seven investigated plant species. One full chloroform extract sample consisted of five cuticular membranes (two for *Hedera helix*) dispersed in the solvent overnight. Samples of methanol extracts were prepared by extracting five CMs overnight. Subsequent chloroform extracts were prepared by dispersing the methanol-extracted membranes in chloroform overnight. Each value represents the mean value ± SD (n = 4 – 8).

Compound class	Carbon chain length	Coverage (μg cm^{-2})		
		Methanol extract	Chloroform extract	Full chloroform extract
Camellia sinensis				
n-alkanes	25	0.03 ± 0.01	0.02 ± 0.01	0.03 ± 0.01
	26	0.01 ± 0.01	0.02 ± 0.01	0.02 ± 0.00
	27	0.05 ± 0.01	0.09 ± 0.01	0.08 ± 0.01
	28	0.01 ± 0.02	0.04 ± 0.01	0.01 ± 0.02
	29	0.18 ± 0.03	0.63 ± 0.03	0.60 ± 0.10
	30	0.01 ± 0.02	0.02 ± 0.01	0.01 ± 0.01
	31	0.03 ± 0.03	0.17 ± 0.01	0.17 ± 0.03
	32	0.04 ± 0.05	0.03 ± 0.01	0.01 ± 0.02
	33		0.05 ± 0.02	
	34		0.04 ± 0.02	
	35		0.04 ± 0.03	
	36		0.03 ± 0.02	
	37		0.02 ± 0.02	
	38		0.02 ± 0.02	
	39		0.02 ± 0.02	
	40		0.01 ± 0.01	
total n-alkanes		*0.36 ± 0.08*	*1.24 ± 0.16*	*0.93 ± 0.14*
primary alcohols	24	0.01± 0.00	0.01 ± 0.01	traces
	25			
	26	0.06± 0.01	0.03 ± 0.01	0.07 ± 0.02
	27	0.02± 0.00	traces	0.01 ± 0.01
	28	0.29± 0.03	0.15 ± 0.02	0.17 ± 0.05
	29	0.06± 0.09	0.04 ± 0.02	0.02 ± 0.03
	30	0.15± 0.15	0.18 ± 0.03	0.65 ± 0.32
	31		0.02 ± 0.00	0.10 ± 0.10
	32		0.11 ± 0.02	0.52 ± 0.19
	34		traces	

total primary alcohols		*0.59 ± 0.10*	*0.55 ± 0.09*	*1.54 ± 0.31*
alkanals	26	traces	traces	
	28	0.03 ± 0.02	0.07 ± 0.08	
	30		0.10 ± 0.10	
	31		traces	
	32		0.08 ± 0.10	
	45			traces
total alkanals		*0.04 ± 0.03*	*0.42 ± 0.18*	*0.02 ± 0.04*
alkanoic acids	24	0.02 ± 0.02	traces	0.02 ± 0.03
	26	0.06 ± 0.06	traces	0.03 ± 0.04
	27			traces
	28	0.09 ± 0.08	0.02 ± 0.03	0.06 ± 0.10
	30	0.09 ± 0.09	traces	0.02 ± 0.04
	32			traces
total alkanoic acids		*0.25 ± 0.25*	*0.05 ± 0.08*	*0.30 ± 0.24*
alkyl esters	22	traces		
	23	traces	traces	
total alkyl esters		*traces*	*traces*	
coumaric acid ester	24	traces		
	26	0.01 ± 0.01	0.04 ± 0.03	0.04 ± 0.04
	28	0.04 ± 0.02	0.03 ± 0.02	0.06 ± 0.11
	30	0.02 ± 0.02	0.04 ± 0.02	0.07 ± 0.12
	32	0.02 ± 0.02	0.06 ± 0.04	
total coumaric acid ester		*0.09 ± 0.06*	*0.17 ± 0.10*	*0.17 ± 0.25*
total very-long-chain aliphatics		*1.34 ± 0.25*	*2.36 ± 0.26*	*2.96 ± 0.34*
Canophyllal				0.23 ± 0.40
Camposterol			0.01 ± 0.02	
Epifridelinol				0.10 ± 0.18
Erythrodiol		0.04 ± 0.07		
Fridelin		2.20 ± 0.09		2.42 ± 0.59
Lupenon				0.01 ± 0.02
Lupeol		0.04 ± 0.05		
Stigmasterol		0.06 ± 0.10	0.01 ± 0.02	
Stigmasterol 5, 24 dien			0.01 ± 0.02	
α-Amyrin		0.01 ± 0.02		0.46 ± 0.16
β-Amyrin		0.06 ± 0.10		0.02 ± 0.04
β-Sitosterol		0.07 ± 0.12	0.08 ± 0.07	
Ursolic acid			0.05 ± 0.09	0.09 ± 0.16
Unspecified		7.25 ± 0.68	0.05 ± 0.06	6.38 ± 0.86
total cyclic aliphatics		*9.74 ± 0.97*	*0.22 ± 0.18*	*9.72 ± 0.79*
Not identified		*0.63 ± 0.29*	*0.41 ± 0.18*	*0.76 ± 0.26*
Total wax		*11.7 ± 1.10*	*3.05 ± 0.16*	*13.4 ± 1.19*

		Ficus elastica		
n-alkanes	25	0.02 ± 0.02	0.09 ± 0.07	0.05 ± 0.04
	26		traces	
	27	0.02 ± 0.03	0.20 ± 0.8	0.31 ± 0.02
	28	0.01 ± 0.01	0.02 ± 0.02	0.05 ± 0.04
	29	traces	0.19 ± 0.15	0.22 ± 0.07
	30		0.04 ± 0.02	0.04 ± 0.05
	31	0.09 ± 0.22	0.41 ± 0.28	0.48 ± 0.14
	32		0.05 ± 0.04	0.18 ± 0.24
	33		0.26 ± 0.20	
total n-alkanes		*0.14 ± 0.20*	*1.28 ± 0.64*	*1.33 ± 0.42*
primary alcohols	24		0.02 ± 0.01	0.02 ± 0.02
	26		0.04 ± 0.02	0.08 ± 0.04
	27		traces	
	28	0.06 ± 0.15	0.09 ± 0.07	0.22 ± 0.05
	29		0.03 ± 0.02	
	30	0.21 ± 0.27	0.12 ± 0.14	
	31		0.09 ± 0.05	0.44 ± 0.33
	32		0.20 ± 0.14	0.32 ± 0.26
	33		0.08 ± 0.06	0.13 ± 0.11
	34		0.13 ± 0.10	0.22 ± 0.10
total primary alcohols		*0.27 ± 0.26*	*0.80 ± 0.54*	*1.42 ± 0.54*
alkanals	28		0.05 ± 0.05	0.05 ± 0.05
	29			0.27 ± 0.39
	30		0.20 ± 0.19	
	31		0.03 ± 0.02	0.02 ± 0.06
	32	0.39 ± 0.96	0.85 ± 0.49	1.32 ± 0.60
	33		0.07 ± 0.07	
	34	0.32 ± 0.79	0.34 ± 0.16	1.13 ± 0.76
	35		0.02 ± 0.03	
total alkanals		*0.71 ± 1.75*	*1.54 ± 0.79*	*2.53 ± 1.27*
alkanoic acids	20	traces		0.02 ± 0.03
	21			
	22	traces		0.01 ± 0.01
	24	0.03 ± 0.03	0.04 ± 0.02	0.10 ± 0.04
	25			traces
	26	0.02 ± 0.03	0.07 ± 0.04	0.19 ± 0.08
	27		traces	
	28	0.12 ± 0.14	0.12 ± 0.09	0.51 ± 0.27
	29		0.05 ± 0.05	0.43 ± 0.34
	30		0.19 ± 0.19	0.37 ± 0.27
	31		0.2 ± 0.05	0.22 ± 0.18
	32	0.10 ± 0.25	0.39 ± 0.44	0.90 ± 0.63
	33		0.03 ± 0.04	
	34		0.17 ± 0.26	0.38 ± 0.34

total alkanoic acids		*0.94 ± 1.00*	*1.12 ± 1.08*	*3.13 ± 1.69*
alkyl esters	24			
	25			0.03 ± 0.08
	26		0.04 ± 0.10	
	27		traces	
	28		0.04 ± 0.10	
	29		0.24 ± 0.41	
	31		0.12 ± 0.25	
	33			0.19 ± 0.30
	36		0.03 ± 0.09	
	Unidentified chain length			0.45 ± 0.19
total alkyl esters			*0.59 ± 0.19*	*0.92 ± 0.66*
total very-long-chain aliphatics		*2.09 ± 2.91*	*5.34 ± 2.47*	*9.32 ± 3.38*
α-Amyrin		0.17 ± 0.20		0.13 ± 0.17
β-Amyrin		0.64 ± 0.75		0.47 ± 0.62
δ-Amyrin		0.08 ± 0.09		0.11 ± 0.19
Fridelin		14.3 ± 1.13	0.09 ± 0.15	15.3 ± 0.09
Fridelinol		3.15 ± 0.90	0.03 ± 0.08	3.49 ± 0.95
Lanosterol		8.72 ± 3.43	traces	8.04 ± 1.97
Lupeol		5.08 ± 2.43		6.57 ± 0.69
β-Sitosterol			0.03 ± 0.05	
Taraxerol		0.19 ± 0.22		0.02 ± 0.06
Unspecified		12.4 ± 8.25	0.16 ± 0.16	14.2 ± 8.36
total cyclic aliphatics		*45.6 ± 3.27*	*0.47 ± 0.45*	*48.3 ± 8.10*
Not identified		*0.44 ± 0.25*	*0.24 ± 0.18*	*0.76 ± 0.84*
Total wax		*48.0 ± 5.10*	*6.05 ± 2.46*	*58.4 ± 10.2*
Hedera helix				
n-alkanes	25	traces	traces	0.06 ± 0.05
	26			0.04 ± 0.03
	27	traces	0.11 ± 0.03	0.10 ± 0.06
	28		0.03 ± 0.04	0.07 ± 0.07
	29	0.11 ± 0.06	0.77 ± 0.08	0.84 ± 0.20
	30	traces	traces	0.06 ± 0.06
	31	0.16 ± 0.17	0.32 ± 0.16	0.28 ± 0.19
	32	traces	0.02 ± 0.04	0.04 ± 0.05
	33		0.08 ± 0.11	
	34			0.03 ± 0.04
	35			0.03 ± 0.03
	36			traces
total n-alkanes		*0.31 ± 0.27*	*1.29 ± 0.39*	*1.57 ± 0.64*
	22	0.06 ± 0.01	0.04 ± 0.01	0.08 ± 0.04
	23	traces		traces
	24	0.11 ± 0.02	0.12 ± 0.02	0.26 ± 0.08

pirmary alcohols	25	traces	traces	0.04 ± 0.01
	26	0.18 ± 0.02	0.31 ± 0.08	0.73 ± 0.14
	27		traces	0.04 ± 0.01
	28	0.14 ± 0.04	0.35 ± 0.12	0.96 ± 0.16
	29		0.08 ± 0.03	0.07 ± 0.05
	30	0.13 ± 0.01	0.50 ± 0.20	1.57 ± 0.41
	31		traces	0.11 ± 0.03
	32	0.04 ± 0.01	0.22 ± 0.05	0.57 ± 0.13
	33			traces
	34		traces	0.05 ± 0.03
total primary alcohols		*0.67 ± 0.10*	*1.67 ± 0.51*	*4.50 ± 0.72*
alkanals	24			0.05 ± 0.03
	25			traces
	26		0.09 ± 0.07	0.14 ± 0.04
	27			traces
	28		0.14 ± 0.11	0.19 ± 0.05
	29			0.03 ± 0.03
	30		0.28 ± 0.09	0.56 ± 0.19
	31			0.03 ± 0.03
	32		0.12 ± 0.07	0.22 ± 0.09
	35			
total alkanals			*0.63 ± 0.30*	*1.25 ± 0.41*
alkanoic acids	20			0.04 ± 0.02
	21			traces
	22	0.05 ± 0.01	0.05 ± 0.03	0.11 ± 0.05
	23	0.02 ± 0.00		0.03 ± 0.02
	24	0.11 ± 0.02	0.14 ± 0.08	0.32 ± 0.10
	25	traces		0.04 ± 0.02
	26	0.08 ± 0.01	0.14 ± 0.09	0.29 ± 0.07
	27		traces	0.05 ± 0.01
	28	0.05 ± 0.02	0.17 ± 0.12	0.29 ± 0.08
	29		0.07 ± 0.04	0.13 ± 0.04
	30	0.08 ± 0.02	0.37 ± 0.27	0.58 ± 0.21
	31		traces	0.06 ± 0.06
	32	traces	0.13 ± 0.13	0.21 ± 0.17
total alkanoic acids		*0.45 ± 0.10*	*1.28 ± 0.53*	*2.16 ± 0.72*
alkyl esters	23		0.11 ± 0.10	
	25		traces	
	26		traces	
	29			traces
	32		0.07 ± 0.12	traces
	34			traces
	36			traces
	37		traces	
	38			0.03 ± 0.03

	40			0.05 ± 0.05
	42		0.08 ± 0.06	0.09 ± 0.04
	44		0.19 ± 0.12	0.10 ± 0.07
	46		0.16 ± 0.10	0.19 ± 0.08
	48		0.11 ± 0.07	0.13 ± 0.04
	50			0.10 ± 0.03
	Unidentified chain length	0.04 ± 0.03	0.36 ± 0.24	0.34 ± 0.24
total alkyl esters		*0.04 ± 0.03*	*1.46 ± 0.24*	*1.16 ± 0.37*
coumaric acid ester	20	0.32 ± 0.04		0.34 ± 0.23
	21	traces		
	22	0.23 ± 0.01	0.04 ± 0.03	0.24 ± 0.12
	23	traces		
	24	0.17 ± 0.03	0.13 ± 0.08	0.26 ± 0.07
	26	0.03 ± 0.02	0.06 ± 0.09	0.09 ± 0.02
	28	traces	0.04 ± 0.04	0.06 ± 0.05
	30		0.11 ± 0.12	0.13 ± 0.07
	32			0.05 ± 0.05
total coumaric acid ester		*0.78 ± 0.10*	*0.44 ± 0.24*	*1.18 ± 0.37*
total very-long-chain aliphatics		*2.24 ± 0.54*	*6.77 ± 1.64*	*11.8 ± 1.17*
Stigmasterol			traces	
β-Sitosterol		traces		
unspecified		0.16 ± 0.04		0.29 ± 0.32
total cyclic aliphatics		*0.18 ± 0.06*	*traces*	*0.29 ± 0.32*
Not identified		*0.56 ± 0.11*	*0.79 ± 0.21*	*0.55 ± 0.60*
Total wax		*2.98 ± 0.52*	*7.58 ± 1.48*	*12.7 ± 2.01*
Ilex aquifolium				
n-alkanes	27			0.01 ± 0.03
	28			
	29		0.08 ± 0.02	0.11 ± 0.04
	30			
	31		0.19 ± 0.03	0.22 ± 0.07
total n-alkanes			0.27 ± 0.02	0.34 ± 0.13
	26		0.06 ± 0.02	0.11 ± 0.04
	29	0.49 ± 0.14		0.29 ± 0.07
	32			0.12 ± 0.17
total alkanoic acids		*0.49 ± 0.14*	*0.06 ± 0.02*	*0.53 ± 0.14*
total very-long-chain aliphatics		*0.49 ± 0.14*	*0.35 ± 0.06*	*0.87 ± 0.12*
α-Amyrin		8.58 ± 2.44		4.89 ± 3.50
β-Amyrin		2.01 ± 0.49		3.42 ± 4.29
δ-Amyrin				1.37 ± 0.79
Betulinic acid		3.64 ± 0.41		3.02 ± 0.71
Erythrodiol		0.86 ± 0.12		0.97 ± 0.45

Hederagenin		0.89 ± 0.13		1.03 ± 0.19
Lupeol		1.67 ± 0.34		1.64 ± 0.29
Oleanolic acid		19.1 ± 2.24	0.03 ± 0.05	18.3 ± 1.95
β-Sitosterol		0.09 ± 0.16		
Ursolic acid		91.8 ± 9.91	0.19 ± 0.34	87.7 ± 12.7
Uvaol		4.15 ± 0.73		4.74 ± 1.11
unspecified		15.5 ± 1.44	0.14 ± 0.20	16.5 ± 2.63
total cyclic aliphatics		*148 ± 15.1*	*0.37 ± 0.35*	*144 ± 16.3*
Not identified		*4.46 ± 1.45*	*0.03 ± 0.06*	*4.18 ± 1.33*
Total wax		*153 ± 14.0*	*0.75 ± 0.38*	*149 ± 16.2*
		Nerium oleander		
n-alkanes	29	0.21 ± 0.07	0.27 ± 0.10	0.41 ± 0.11
	30	0.05 ± 0.09	0.09 ± 0.03	0.09 ± 0.07
	31	0.49 ± 0.43	0.78 ± 0.30	1.00 ± 0.25
	32		0.14 ± 0.05	0.19 ± 0.04
	33		0.87 ± 0.37	1.18 ± 0.20
	34		0.08 ± 0.09	
	35	2.30 ± 0.79	0.77 ± 0.25	2.10 ± 1.45
	36		0.16 ± 0.09	
	37	0.40 ± 0.42	0.58 ± 0.17	0.47 ± 0.09
	39			0.10 ± 0.10
total n-alkanes		*3.45 ± 0.85*	*3.74 ± 1.33*	*5.55 ± 1.74*
pirmary alcohols	29	0.15 ± 0.14	0.11 ± 0.04	0.13 ± 0.04
	30	0.17 ± 0.18	0.03 ± 0.04	0.21 ± 0.14
	31		0.18 ± 0.07	
	34	0.29 ± 0.31	0.19 ± 0.07	0.39 ± 0.32
	36			0.06 ± 0.11
total primary alcohols		0.62 ± 0.48	0.52 ± 0.16	0.79 ± 0.28
alkanoic acids	30	0.22 ± 0.38		0.07 ± 0.13
	32			0.24 ± 0.41
total alkanoic acids		0.45 ± 0.10	1.28 ± 0.53	2.16 ± 0.72
total very-long-chain aliphatics		4.29 ± 1.37	4.39 ± 1.43	6.65 ± 1.71
α-Amyrin		0.14 ± 0.10		0.13 ± 0.04
Betulinic acid		1.67 ± 1.70		0.91 ± 1.57
Erythrodiol		0.39 ± 0.25		0.70 ± 0.63
Hederagenin		0.63 ± 0.24		1.18 ± 0.69
Oleanolic acid		40.6 ± 4.37		42.5 ± 9.59
Ursolic acid		123 ± 18.1	0.04 ± 0.07	126 ± 43.8
Uvaol				0.55 ± 0.95
unspecified		15.6 ± 1.79	0.07 ± 0.07	17.9 ± 0.71
total cyclic aliphatics		*188 ± 26.4*	*0.16 ± 0.04*	*190 ± 52.0*
Not identified		*2.18 ± 1.46*	*0.06 ± 0.09*	*1.80 ± 0.53*

Total wax		*188 ± 26.4*	*4.61 ± 1.50*	*198 ± 53.9*
		Vinca minor		
n-alkanes	25	traces	traces	
	26	traces		
	27	traces	traces	0.02 ± 0.01
	28	traces	0.06 ± 0.02	
	29	0.05 ± 0.05	0.07 ± 0.01	0.14 ± 0.14
	30	traces	0.04 ± 0.02	0.02 ± 0.01
	31	0.09 ± 0.11	0.29 ± 0.03	0.56 ± 0.32
	32			0.07 ± 0.01
	33		0.27 ± 0.04	0.34 ± 0.04
total n-alkanes		*0.19 ± 0.24*	*0.77 ± 0.09*	*1.16 ± 0.42*
pirmary alcohols	24		0.04 ± 0.01	0.05 ± 0.00
	25			0.02 ± 0.00
	26	0.02 ± 0.00	0.12 ± 0.02	0.18 ± 0.01
	27		traces	0.05 ± 0.01
	28	0.05 ± 0.01	0.14 ± 0.03	0.23 ± 0.02
	29	0.03 ± 0.02		0.06 ± 0.00
	30		0.08 ± 0.05	0.18 ± 0.06
	31		0.07 ± 0.04	0.07 ± 0.12
	32		0.16 ± 0.01	
	33		0.08 ± 0.05	0.25 ± 0.05
	34		0.10 ± 0.06	0.20 ± 0.08
total primary alcohols		*0.11 ± 0.02*	*0.80 ± 0.21*	*1.29 ± 0.11*
alkanals	31		0.07 ± 0.04	
	32		0.20 ± 0.14	0.10 ± 0.12
	33		0.06 ± 0.03	
	34		0.17 ± 0.15	0.60 ± 0.15
total alkanals			*0.49 ± 0.34*	*0.70 ± 0.12*
	24			0.03 ± 0.01
	26		traces	0.04 ± 0.00
	27			
	28	0.02 ± 0.04	0.07 ± 0.04	0.08 ± 0.01
	29			traces
	30		0.09 ± 0.05	
	31		0.12 ± 0.07	
	32		0.14 ± 0.09	0.17 ± 0.10
	34			0.13 ± 0.06
total alkanoic acids		*0.02 ± 0.04*	*0.43 ± 0.26*	*0.46 ± 0.11*
alkyl esters	32			*0.03 ± 0.01*
total alkyl esters				*0.03 ± 0.01*
total very-long-chain aliphatics		*0.22 ± 0.04*	*1.65 ± 0.93*	*3.17 ± 0.30*
α-Amyrin		traces		0.04 ± 0.00
Erythrodiol		0.08 ± 0.01		0.24 ± 0.07

Oleanolic acid		7.10 ± 0.99		6.88 ± 0.33
Ursolic acid		32.3 ± 8.80		27.0 ± 2.85
Uvaol		1.44 ± 0.06		1.63 ± 0.10
Unspecified		3.58 ± 0.30	0.04 ± 0.06	3.49 ± 0.29
total cyclic aliphatics		*44.5 ± 40.1*	*0.04 ± 0.06*	*39.3 ± 2.75*
Not identified		*0.26 ± 0.03*	*0.08 ± 0.06*	*0.86 ± 0.34*
Total wax		*45.1 ± 10.4*	*2.61 ± 0.60*	*43.8 ± 2.66*
		Zamioculcas zamiifolia		
n-alkanes	25	traces	0.03 ± 0.01	0.04 ± 0.01
	26	traces	traces	
	27	traces	0.06 ± 0.01	0.06 ± 0.01
	28	traces	traces	
	29	0.04 ± 0.05	0.09 ± 0.03	0.06 ± 0.01
	30	traces	0.02 ± 0.01	
	31	0.08 ± 0.03	0.25 ± 0.14	0.19 ± 0.03
	32		traces	
total n-alkanes		*0.14 ± 0.08*	*0.47 ± 0.20*	*0.34 ± 0.04*
pirmary alcohols	24	0.03 ± 0.01	0.03 ± 0.01	0.05 ± 0.01
	25	0.16 ± 0.10	traces	
	26	traces	0.52± 0.18	0.70 ± 0.06
	27		0.03 ± 0.01	0.06 ± 0.01
	28	0.25 ± 0.09	1.50± 0.41	1.76 ± 0.12
	29		0.04 ± 0.01	
	30	0.08 ± 0.02	0.68 ± 0.15	0.72 ± 0.07
	31		traces	
	32	0.09 ± 0.04	0.66 ± 0.15	0.59 ± 0.10
	33		traces	
	34		0.09 ± 0.02	0.05 ± 0.03
total primary alcohols		*0.62 ± 0.21*	*3.60 ± 0.61*	*3.89 ± 0.35*
alkanals	27		0.04 ± 0.01	
	28		0.04 ± 0.01	
	29		0.09 ± 0.06	
	30		traces	
	31		traces	
	32		0.12 ± 0.10	
total alkanals			*0.30 ± 0.08*	
alkanoic acids	20	0.03 ± 0.01	0.01 ± 0.00	0.06 ± 0.01
	24	0.03 ± 0.02	0.03 ± 0.02	0.06 ± 0.01
	25		traces	
	26	0.12 ± 0.06	0.54 ± 0.35	0.69 ± 0.22
	27		0.04 ± 0.01	
	28	0.10 ± 0.07	1.51 ± 0.93	1.10 ± 0.59
	29		0.22 ± 0.29	
	30	0.03 ± 0.02	1.09 ± 0.29	0.30 ± 0.20
	31		0.17 ± 0.12	

	32		0.16 ± 0.08	traces
total alkanoic acids		*0.31 ± 0.16*	*3.58 ± 1.36*	*2.25 ± 1.04*
alkyl esters	26		traces	
	27		traces	
	33		0.06 ± 0.10	
	34		0.10 ± 0.17	
	42		0.16 ± 0.10	0.24 ± 0.01
	44		0.29 ± 0.18	0.37 ± 0.02
	46		0.11 ± 0.07	0.13 ± 0.01
	48		traces	
total alkyl esters			*0.75 ± 0.23*	*0.78 ± 0.03*
total very-long-chain aliphatics		*1.07 ± 0.31*	*8.70 ± 1.51*	*7.27 ± 0.93*
Camposterol		0.03 ± 0.02		0.04 ± 0.02
Oleanoic acid				0.07 ± 0.12
β-Sitosterol		traces		
Stigmasterol		traces		
δ-Tocopherol		traces		traces
Ursolic acid		0.06 ± 0.10		0.04 ± 0.06
Unspecified				0.07 ± 0.05
total cyclic aliphatics		*0.12 ± 0.12*		*0.24 ± 0.23*
Not identified		*0.06 ± 0.04*	*0.12 ± 0.01*	*traces*
Total wax		*1.26 ± 0.30*	*8.81 ± 1.93*	*7.53 ± 0.85*

Supplementary Table 2. Triterpenoid and very long-chain aliphatic loads extracted with methanol and subsequent chloroform, and full chloroform extracts of seven plant species. Each value represents the mean value ± SD (*n* = 4 – 8).

Plant Species	Coverage (μg cm^{-2})							
	Triterpenoid content				Very long-chain aliphatic content			
	Methanol extract	Chloroform extract	Accumulated extract	Full chloroform extract	Methanol extract	Chloroform extract	Accumulated extract	Full chloroform extract
Camellia sinensis	9.74 ± 0.97	0.22 ± 0.18	9.96 ± 1.09	9.72 ± 0.79	1.34 ± 0.25	2.36 ± 0.26	3.70 ± 0.45	2.96 ± 0.34
Ficus elastica	45.5 ± 3.27	0.47 ± 0.45	45.9 ± 3.62	48.3 ± 8.10	2.09 ± 2.91	5.34 ± 2.47	7.42 ± 3.26	9.32 ± 3.3
Hedera helix	0.18 ± 0.06	0.02 ± 0.03	0.20 ± 0.05	0.29 ± 0.32	2.24 ± 0.54	6.77 ± 1.64	9.01 ± 1.65	11.8 ± 1.16
Ilex aquifolium	148 ± 15.1	0.37 ± 0.35	149 ± 14.8	144 ± 16.3	0.50 ± 0.14	0.35 ± 0.06	0.85 ± 0.10	0.87 ± 0.12
Nerium oleander	182 ± 26.0	0.16 ± 0.04	183 ± 26.8	190 ± 52.0	4.29 ± 1.37	4.39 ± 1.43	7.88 ± 2.63	6.65 ± 1.71
Vinca minor	44.5 ± 10.1	0.04 ± 0.06	44.6 ± 10.20	39.3 ± 2.75	0.32 ± 0.24	2.50 ± 0.72	2.82 ± 0.80	3.64 ± 0.46
Zamioculcas zamiifeia	0.12 ± 0.12	0.00 ± 0.00	0.12 ± 0.12	0.24 ± 0.23	1.07 ± 0.31	8.70 ± 1.51	9.77 ± 2.19	7.27 ± 0.93

Supplementary Table 3. Water permeability and effect on the permeance barrier of stomata free cuticular leaf discs of seven plant species after different treatments in untreated, methanol extracted and dewaxed cuticles. The latter were prepared by extracting CMs overnight with methanol and subsequently with chloroform overnight. Each value represents the median and 25th - 75th percentile (n > 28).

Plant Species	Permeance			Effect on permeance	
	Untreated cuticle x 10^{-5} (m s^{-1})	Methanol extracted cuticle x 10^{-5}(m s^{-1})	Dewaxed cuticle x 10^{-5} (m s^{-1})	Effect on permeance of methanol extracted cuticles	Effect on permeance of dewaxed cuticles
Camellia sinensis	4.27, 1.49 - 7.60	1.23, 0.67 - 5.49	24.9,18.9 – 35.6	0.5, 0.3 - 0.8	7.5, 4.5 - 13
Ficus elastica	0.97, 0.30 - 3.01	1.44, 0.15 - 8.23	5.39, 3.59 – 10.6	1.3, 0. 8 - 2.2	7.3, 4.3 - 12
Hedera helix	0.40, 0.26 – 0.79	0.69, 0.28 - 1.31	7.60, 2.37 – 18.4	1.3, 1.0 - 2.2	17, 11 - 24
Ilex aquifolium	1.31, 0.41 - 3.66	2.03, 1.45 - 5.52	30.9, 26.2 – 38.3	2.0, 1. 5 - 2.8	27, 19 - 38
Nerium oleander	2.14, 1.30 - 3.23	9.19, 5.51 – 15.2	41.0, 33.9 – 48.3	5.0, 4.0 - 6.2	221, 7 - 28
Vinca minor	1.24, 0.77 - 2.10	6.57, 4.78 – 11.5	50.9, 37.0 – 65.0	6.3, 5.2 - 7.6	37, 30 - 45
Zamioculcas zamiifeia	0.45, 0.14 - 1.22	0.57, 0.14 - 2.07	1.07, 0.52 - 2.85	1.1, 0.7 - 1.8	3.3, 2.1 - 5.4

Supplementary Table 4. Weighted average chain length of the extraction with methanol (MeOH), the subsequent chloroform (TCM) extract, the combined MeOH and following TCM extracts and full extracts (FE) of seven investigated plant species. Values show mean ± standard deviation (n = 4 – 8).

Plant Species	Weighted average chain length (number of C atoms)			
	Methanol extract	Chloroform extract	Accumulated extract	Full extract
Camellia sinensis	28.4 ± 0.19	29.9 ± 0.51	29.4 ± 0.38	29.7 ± 0.46
Ficus elastica	27.4 ± 3.24	31.0 ± 0.21	30.8 ± 0.53	30.8 ± 0.58
Hedera helix	25.1 ± 0.65	30.5 ± 0.67	29.1 ± 0.25	29.3 ± 0.29
Ilex aquifolium	28.8 ± 0.44	29.5 ± 0.37	29.0 ± 0.31	29.3 ± 0.71
Nerium oleander	33.8 ± 0.42	33.2 ± 0.10	33.5 ± 0.22	33.1 ± 0.79
Vinca minor	28.6 ± 0.48	30.7 ± 0.62	30.5 ± 0.66	31.2 ± 0.32
Zamioculcas zamiifeia	27.6 ± 0.27	30.1 ± 0.26	29.8 ± 0.29	29.8 ± 0.22

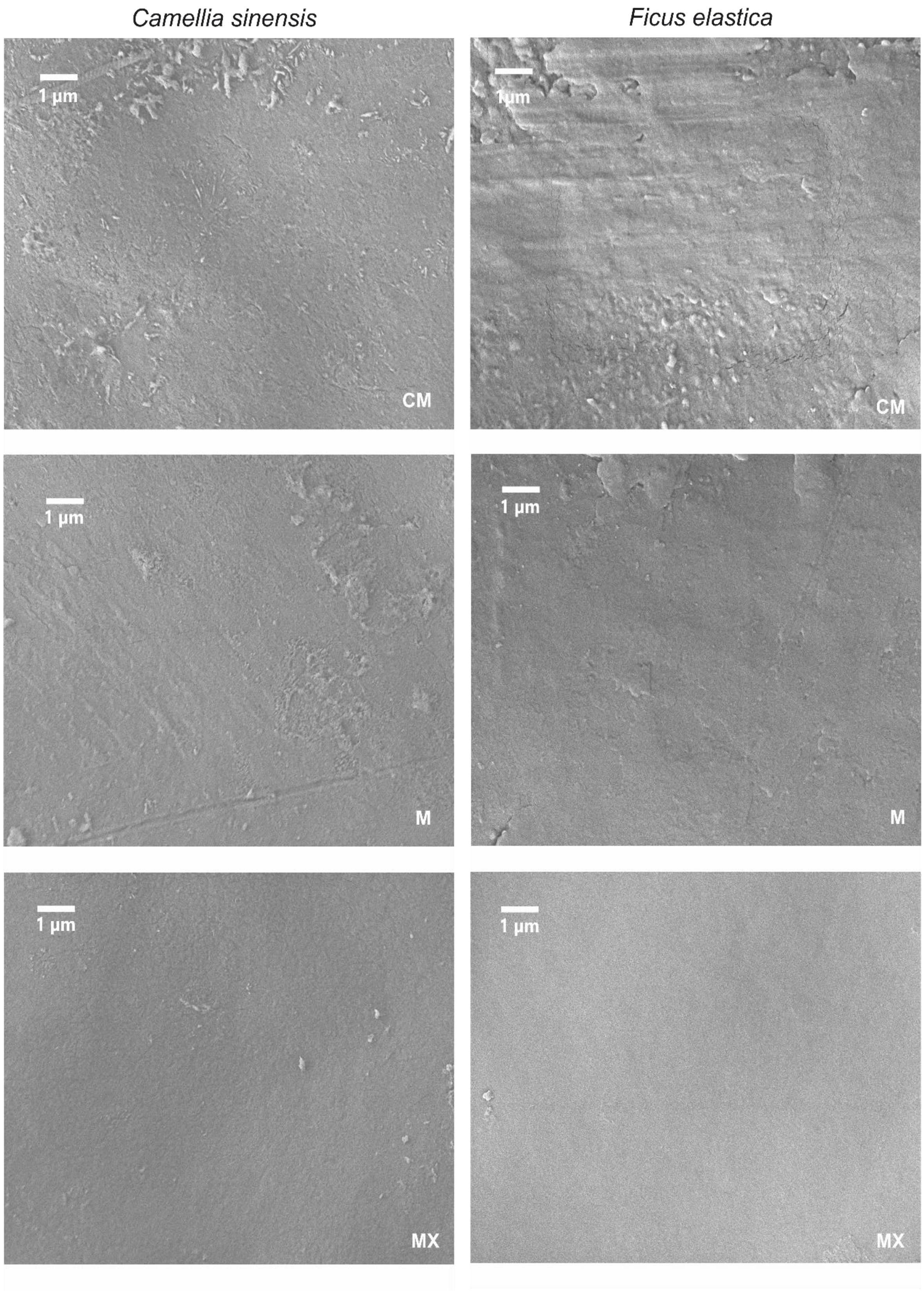

Supplementary Figure 1. Scanning electron microscopy (SEM) images of the outer surface of adaxial isolated cuticular membranes (CM), methanol treated membranes (M) and chloroform treated membranes (MX) of *Camellia sinensis* (left) and *Ficus elastica* (right).

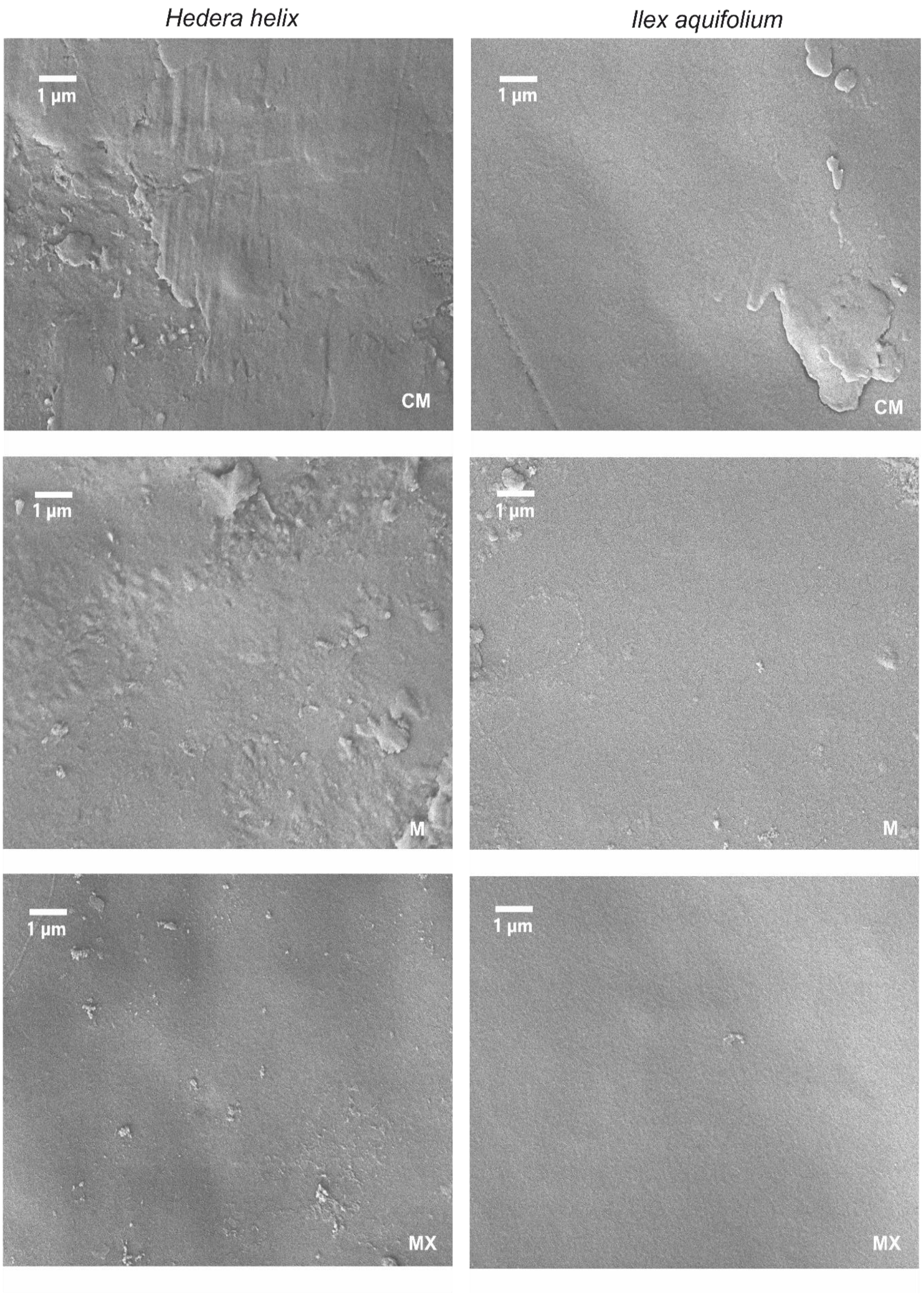

Supplementary Figure 2. Scanning electron microscopy (SEM) images of the outer surface of adaxial isolated cuticular membranes (CM), methanol treated membranes (M) and chloroform treated membranes (MX) of *Hedera helix* (left) and *Ilex aquifolium* (right).

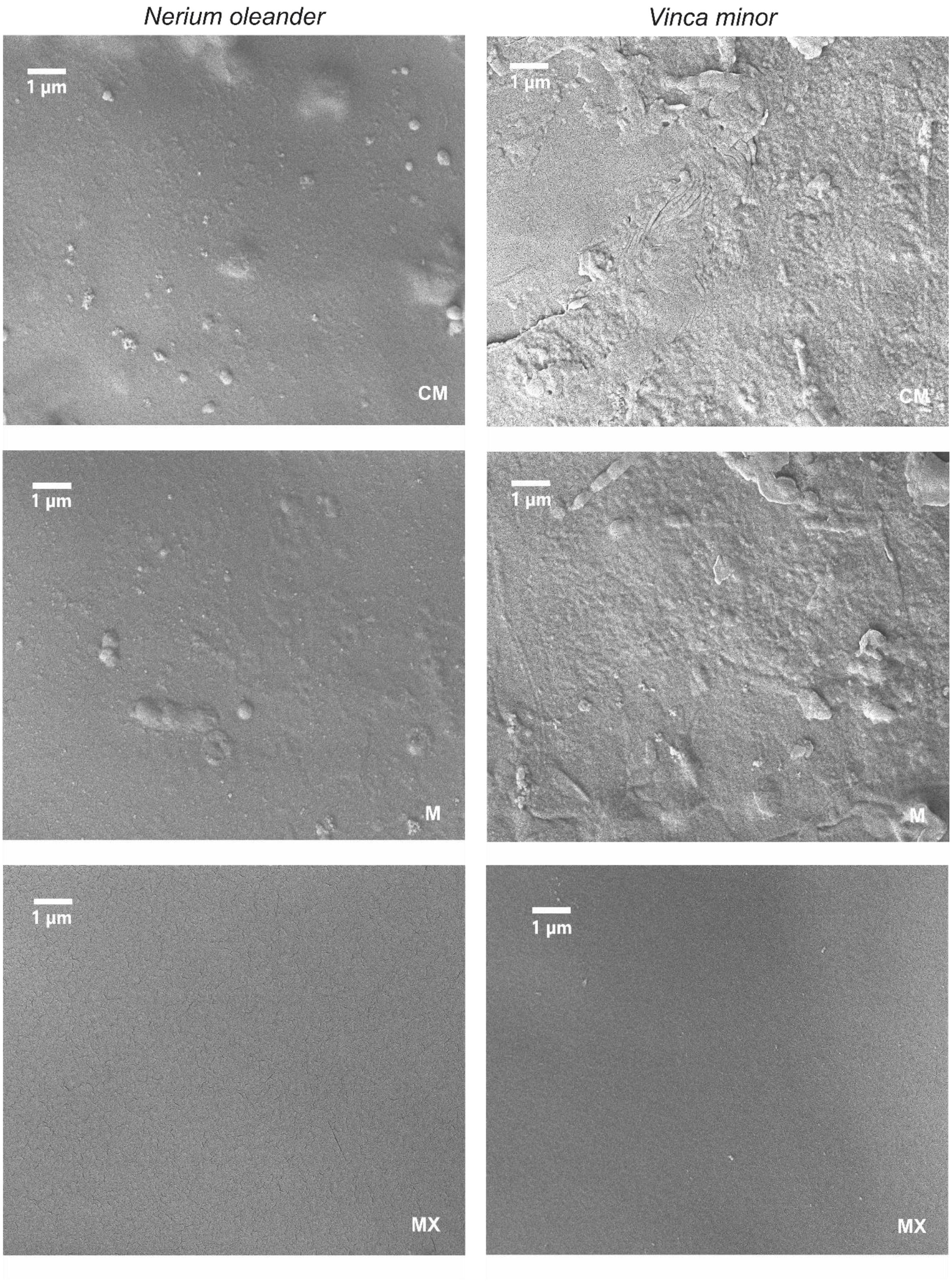

Supplementary Figure 3. Scanning electron microscopy (SEM) images of the outer surface of adaxial isolated cuticular membranes (CM), methanol treated membranes (M) and chloroform treated membranes (MX) of *Nerium oleander* (left) and *Vinca minor* (right).

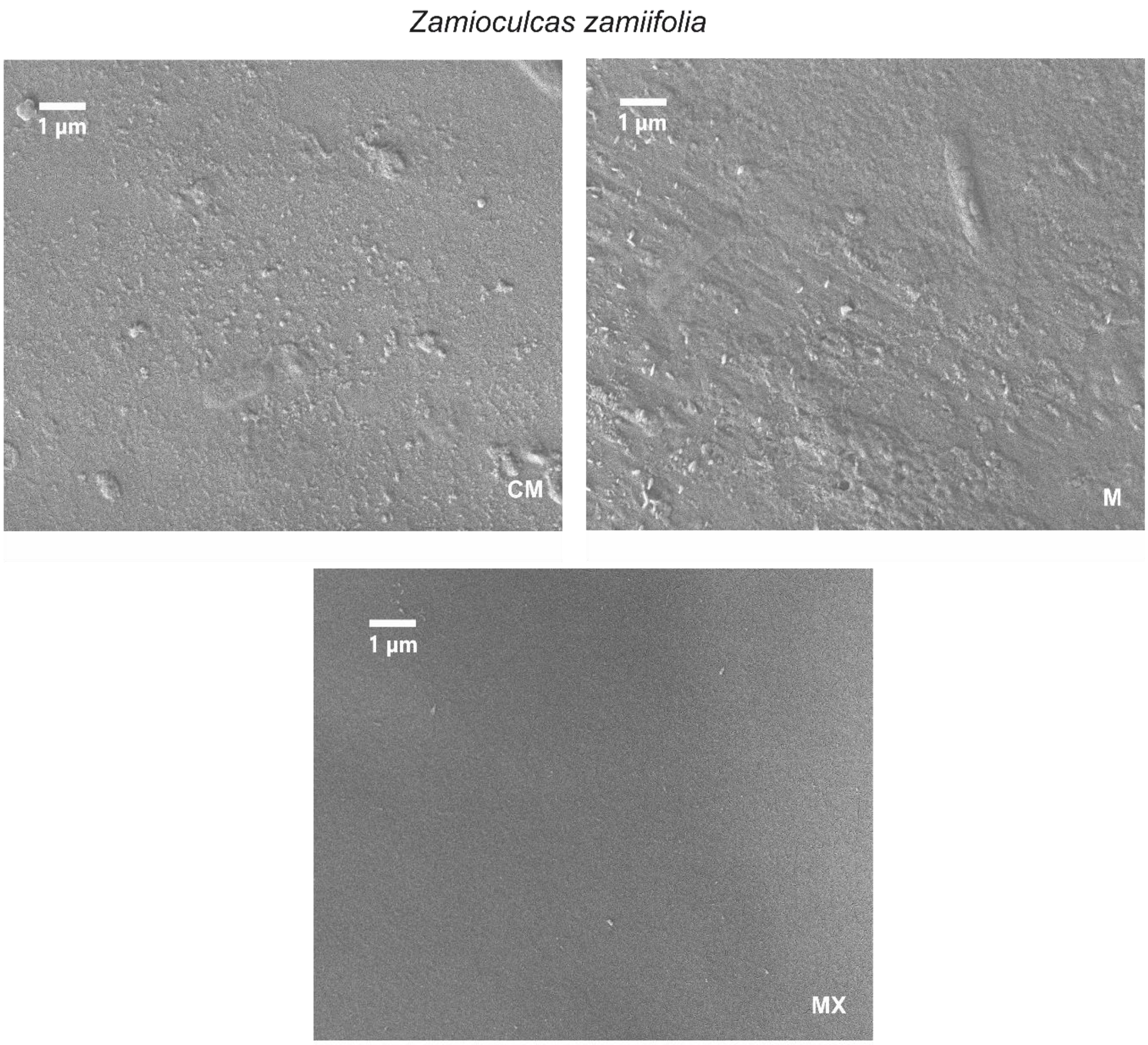

Supplementary Figure 4. Scanning electron microscopy (SEM) images of the outer surface of adaxial isolated cuticular membranes (CM), methanol treated membranes (M) and chloroform treated membranes (MX) of *Zamioculcas zamiifolia*.
